# CacPred: a cascaded convolutional neural network for TF-DNA binding prediction

**DOI:** 10.1186/s12864-025-11399-y

**Published:** 2025-03-18

**Authors:** Shuangquan Zhang, Anjun Ma, Xuping Xie, Zhichao Lian, Yan Wang

**Affiliations:** 1 School of Cyber Science and Engineering, Nanjing University of Science and Technology, Nanjing, 210094 China; 2 Key Laboratory of Symbol Computation and Knowledge Engineering of Ministry of Education, College of Computer Science and Technology, Jilin University, Changchun, 130012 China; 3 Department of Biomedical Informatics, College of Medicine, The Ohio State University, Columbus, OH 43210 USA

**Keywords:** Transcription factor, ChIP-seq, Deep learning, TF-DNA binding prediction

## Abstract

**Background:**

Transcription factors (TFs) regulate the genes’ expression by binding to DNA sequences. Aligned TFBSs of the same TF are seen as cis-regulatory motifs, and substantial computational efforts have been invested to find motifs. In recent years, convolutional neural networks (CNNs) have succeeded in TF-DNA binding prediction, but existing DL methods’ accuracy needs to be improved and convolution function in TF-DNA binding prediction should be further explored.

**Results:**

We develop a cascaded convolutional neural network model named CacPred to predict TF-DNA binding on 790 Chromatin immunoprecipitation-sequencing (ChIP-seq) datasets and seven ChIP-nexus (chromatin immunoprecipitation experiments with nucleotide resolution through exonuclease, unique barcode, and single ligation) datasets. We compare CacPred to six existing DL models across nine standard evaluation metrics. Our results indicate that CacPred outperforms all comparison models for TF-DNA binding prediction, and the average accuracy (ACC), matthews correlation coefficient (MCC), and the area of eight metrics radar (AEMR) are improved by 3.3%, 9.2%, and 6.4% on 790 ChIP-seq datasets. Meanwhile, CacPred improves the average ACC, MCC, and AEMR of 5.5%, 16.8%, and 12.9% on seven ChIP-nexus datasets. To explain the proposed method, motifs are used to show features CacPred learned. In light of the results, CacPred can find some significant motifs from input sequences.

**Conclusions:**

This paper indicates that CacPred performs better than existing models on ChIP-seq data. Seven ChIP-nexus datasets are also analyzed, and they coincide with results that our proposed method performs the best on ChIP-seq data. CacPred only is equipped with the convolutional algorithm, demonstrating that pooling processing of the existing models leads to losing some sequence information. Some significant motifs are found, showing that CacPred can learn features from input sequences. In this study, we demonstrate that CacPred is an effective and feasible model for predicting TF-DNA binding. CacPred is freely available at https://github.com/zhangsq06/CacPred.

**Supplementary Information:**

The online version contains supplementary material available at 10.1186/s12864-025-11399-y.

## Background

Transcription factors (TFs) act as a crucial role in gene expression, cellular processes and transcriptional regulatory networks by binding to transcription factor binding sites (TFBSs) [1, 2]. TF-DNA binding prediction and identifying TFBSs are fundamental challenges for revealing the regulatory mechanisms of TFs and TF’s cooperation [3, 4]. Chromatin immunoprecipitation-sequencing (ChIP-seq) combines ChIP technology and high-throughput sequencing to obtain a TF binding region on genomic sequences. Meanwhile, ChIP-nexus combines exonuclease, specific barcodes, and a single ligation step, and adds an efficient DNA self-circularization step in the library preparation process that achieves a single nucleotide resolution [5]. TFBSs are short and conserved sequences, which increases the difficulty of position prediction via computational methods [6]. The aligned TFBSs are a regulatory motif, which can be represented by the position weight matrix (PWM) [7]. Although some public databases contain documented motifs, lots of unknown motifs and potential TF regulatory mechanisms need to be discovered.

Substantial computational efforts have been invested in predicting TF-DNA binding and finding motifs [8]. For example, MEME-ChIP employed expectation–maximization and DREME to discover Ab initio motifs [9], and gkm-SVM ultilized gap-ker and support vector machine (SVM) to combine multiple similar k-mers into more interpretable PWMs [10]. Because of the complexity of the TF binding mechanisms and the generation of large-scale genomic sequencing data, these models are difficult to handle large-scale data and reveal complex regulatory mechanisms of TFs.

Deep learning (DL) algorithms including convolutional neural networks (CNNs) [11, 12], recurrent neural networks (RNNs) [13, 14], and deep belief networks (DBNs) [15], have exhibited tremendous progress and obtained record-breaking performance in biological applications including cancer classification [16, 17], protein model quality assessment [18–20], and lesion recognition [21]. Meanwhile, previous research has proved DL is a feasible method for TF-DNA binding prediction and motif finding [20, 22]. DeepBind, a method proposed by Alipanahi et al. in 2015, was the first DL model to utilize a CNN to find DNA motifs [23]. DeepBind employed the convolutional kernels as motif detectors, which gives new insight into motif finding. DeepBind achieved better performance than MEME-ChIP and gkm-SVM [23]. Inspired by DeepBind, more DL models are developed for TF-DNA binding prediction and motif finding, such as DeeperBind, Basset, DeepHistone, and TBiNet, et al*.* Especially, DeeperBind employed CNNs and RNNs, which was developed based on DeepBind model. Among all DL models, a recently developed DL model, named DESSO, is the first model to utilize features of DNA shape (HelT, MGW, ProT, and Roll) and combine the binomial hypothetical test with CNNs to find DNA sequence and shape motifs [20]. Because RNN can capture the information within sequences, RNN is also used to find motifs and predict TF-DNA binding [24–28]. Our previous research assessed 20 DL models across 871 ChIP-seq datasets and defined an area of eight metrics radar (AEMR) score to evaluate the performance of these models [20]. The existing 20 DL models all employed convolutional layer, which demonstrates that convolution plays a critical role in TF-DNA binding predicting and motif finding. Our results indicated that DeepHistone is the top model for sequence classification, and DESSO is the top model for motif finding, respectively [29]. Through the previous research, we found that DL methods have a great advantage over the traditional methods. Meanwhile, we also found that the convolution’s function should be further explored and existing model’s accuracy need to be improved.

This paper proposes a cascaded convolutional neural network (CacPred) for TF-DNA binding prediction. Based on our previous research, six competitive models are selected as comparison models. In addition, evaluation metrics including precision, recall, F1_score, accuracy (ACC), specificity, Matthews correlation coefficient (MCC), area under the receiver operating characteristic curve (AUC) and area under the precision-recall curve (PRC) are used to assess DL models’ performance. Meanwhile, AEMR is selected as an overall score to rank all DL models. First, the CacPred model is assessed on 790 ChIP-seq datasets covering 261 TFs, and CacPred improves the average ACC of 3.3%, MCC of 9.2%, and AEMR of 6.4%, in predicting TF-DNA binding. Then, to verify the generalization of CacPred, CacPred is tested on seven ChIP-nexus datasets and improves the average ACC, MCC, and AEMR of 5.5%, 16.8%, and 12.9%. To explain the CacPred model, motifs are used to represent the features the CacPred learned. Our results demonstrate that motifs CacPred found are significant by comparing them to the motif database (HOCOMOCO.v11).

## Results

### Datasets and preprocessing

The experimental data includes 790 ChIP-seq datasets covering 261 TFs and seven ChIP-nexus datasets (Table. S1) covering seven TFs in this paper [30]. The 790 ChIP-seq datasets contain 690 ENCODE ChIP-seq datasets covering 161 TFs and 100 ChIP-seq datasets of Cistrome database covering 100 TFs [31]. All sequences in each sub-dataset are fixed with 1,001bps around their centers, and ranked in the decreasing order of original signal scores, which are all positive samples with label ‘1’. For a sub-dataset, we define a sequence to be a negative sample, which has matched GC-content to a positive sample and doesn’t overlap with any peaks in positive samples. So, the ratio of positive and negative samples is 1:1. This paper selects negative samples with the 1,001-bp-long sequences from the human genome, which are all negative sample with the label ‘0’. Each negative sequence is labeled as ‘0’, meaning that TFs can't bind to them. CacPred needs two inputs, *i.e.* forward sequence and reverse complementary sequence, each of which must be binary vectors. So, each sequence is encoded as a $M = 4 \times 1001$ matrix, *i.e.* A = [1, 0, 0, 0], G = [0, 1, 0, 0], C = [0, 0, 1, 0], T = [0, 0, 0, 1].

### Experimental setup

**Fig. 1:**
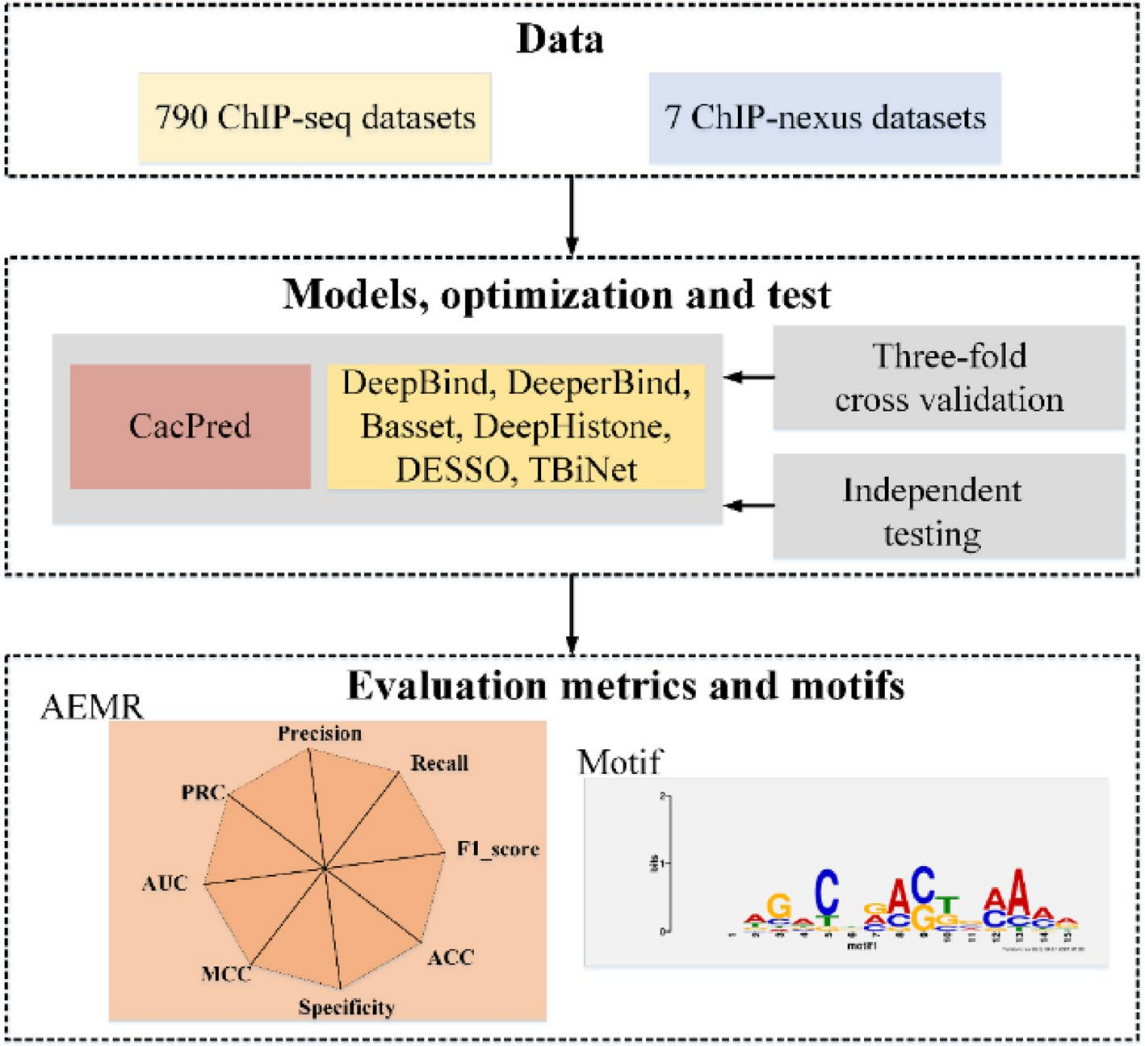
The workflow of the experimental setting

CacPred is optimized to minimize the average loss from the BCEloss by the Adadelta algorithm [32, 33]. To avoid the overfitting issue, dropout is used in CacPred model [34]. For a sub-dataset, 80% of samples are set as a training set, and 20% of samples are set as a testing set. The hyper-parameters contain dropout ratio, batch size, and learning rate in our experiments, which are optimized by three-fold cross-validation on the training set. Epochs of training were set to 20, the AUC of the validation set is calculated. When the AUC is highest in the validation set, hyper-parameters are saved and applied to the testing data. The CacPred model is implemented by Pytorch [35]. This study selects Basset [36], DeepHistone [22], DESSO, DeepBind, DeeperBind, TBiNet [10] as comparison models, based on previous research. The metrics that contain precision, recall, F1_score, ACC, specificity, MCC, AUC, PRC, and AEMR (formula 2) are used to assess models’ performance. To explain CacPred, motifs are used to show features that CacPred learned. The workflow of the experimental setting is shown in Fig. 1.

1 $$O_{i,i + 1} = \frac{1}{2}R_{i} \cdot R_{i + 1} \cdot \sin (\frac{\pi }{4}){\kern 1pt} {\kern 1pt} {\kern 1pt} {\kern 1pt} {\kern 1pt} {\kern 1pt} {\kern 1pt} {\kern 1pt} {\kern 1pt} i = 1,...,8$$

$$R = [precision,recall,F1\_score,ACC,specificity,MCC,AUC,PRC,precision]$$

2 $$AEMR = sum(O_{i,i + 1} ,...){\kern 1pt} {\kern 1pt} {\kern 1pt} {\kern 1pt} {\kern 1pt} {\kern 1pt} {\kern 1pt} i = 1,...,8$$

### Performance on 790 ChIP-seq datasets

**Fig. 2:**
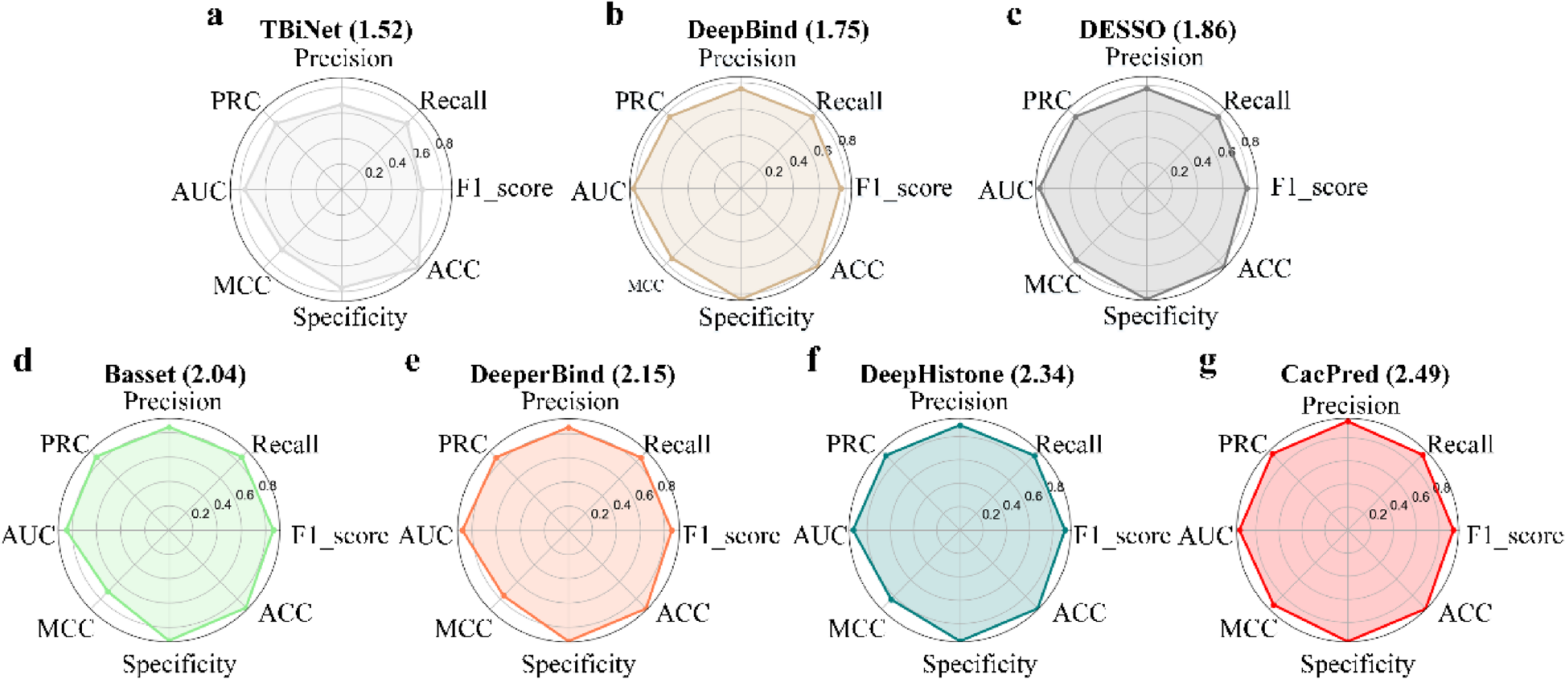
A comparison of CacPred and the comparison models on 790 ChIP-seq datasets across the AEMR metric

CacPred model is compared to six existing models on 790 ChIP-seq datasets. We calculate AEMRs of all models on the testing data and rank them by the average AEMR scores. Figure 2 shows the CacPred model obtains an AEMR score of 2.49 (Fig. 2g), outperforms DeepHistone under the AEMR score, and improves the AEMR score by 6.4%. DeepBind model is the first model to find motifs and predict the TF-DNA binding, which obtains the AEMR of 1.75. TBiNet obtains the AEMR of 1.52, which is lower than DeepBind model.

**Fig. 3:**
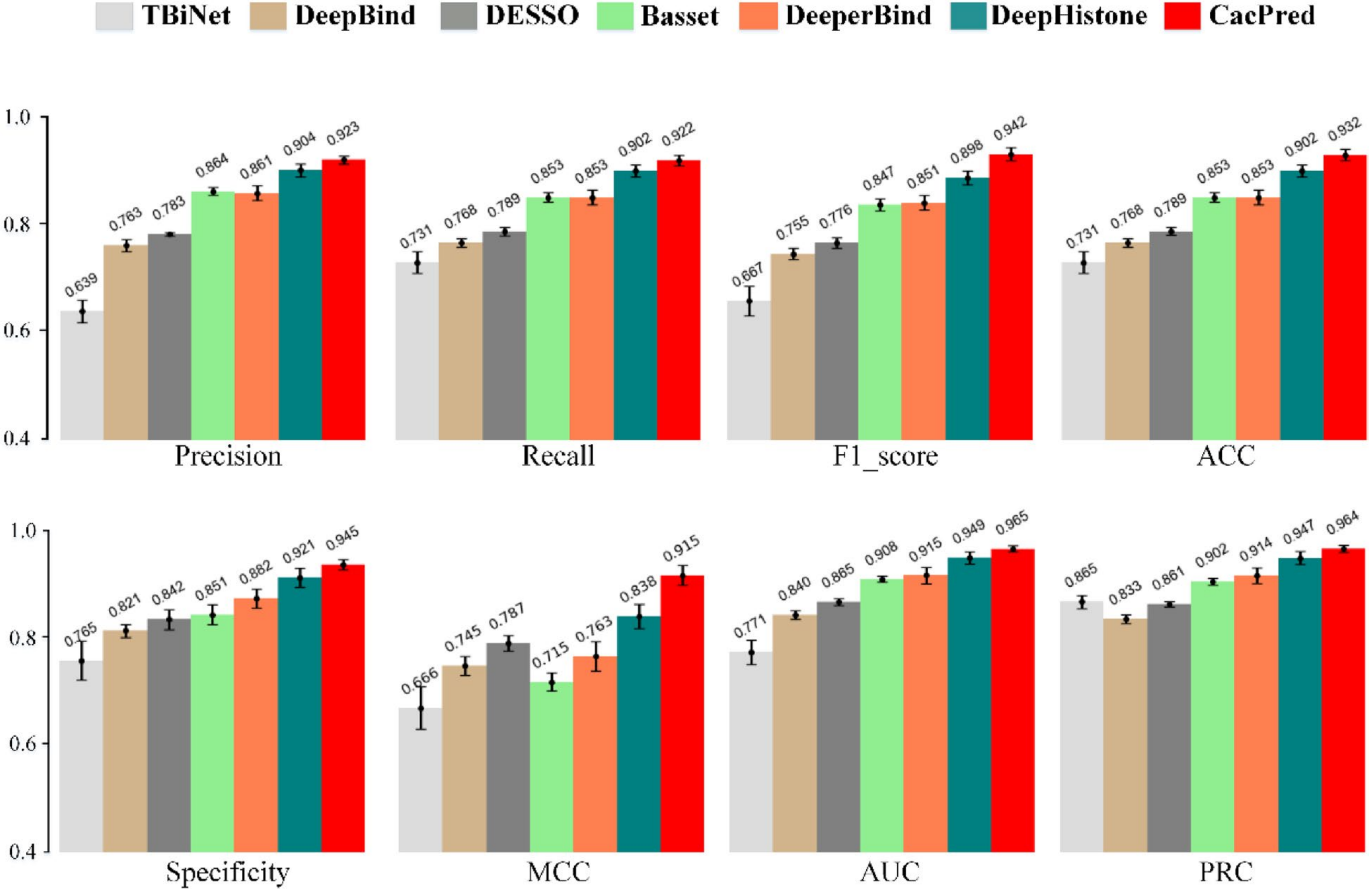
A comparison of CacPred and comparison models on 790 ChIP-seq datasets under the average precision, recall, F1_score, specificity, ACC, MCC, AUC, and PRC metrics

CacPred yields the highest score under precision, recall, F1_score, specificity, ACC, MCC, AUC, and PRC metrics, and achieves the average 0.923, 0.922, 0.942, 0.932, 0.945, 0.915, 0.965, and 0.964 scores, respectively (Fig. 3). And CacPred improves the MCC score of 9.2% in our evaluation. In light of our results, the performance of DL models except DeepBind is consistent. DeepBind yields a higher score than TBiNet under precision, recall, F1_score, specificity, ACC, MCC, and AUC, but the PRC score of DeepBind is lower than TBiNet. Figure 3 also gives the standard deviations (STD) of all models across precision, recall, F1_score, specificity, ACC, MCC, AUC, and PRC. CacPred achieves the lowest STD of precision, recall, F1_score, ACC, MCC, AUC, and PRC than others. Furthermore, TBiNet obtains the highest STD of eight metrics, which demonstrates the stability of TBiNet needs to be improved. Further, this study tries to validate CacPred on cross-cell type TF binding data and select the ChIP-seq data of ETS1 from 690 ENCODE ChIP-seq datasets. The CacPred is trained on K562 of ETS1 and is tested on GM12878 and K549 of ETS1. Our results show that CacPred outperforms the comparison models on the AEMR score (Table. S2).

### Validating CacPred on ChIP-nexus datasets

**Fig. 4:**
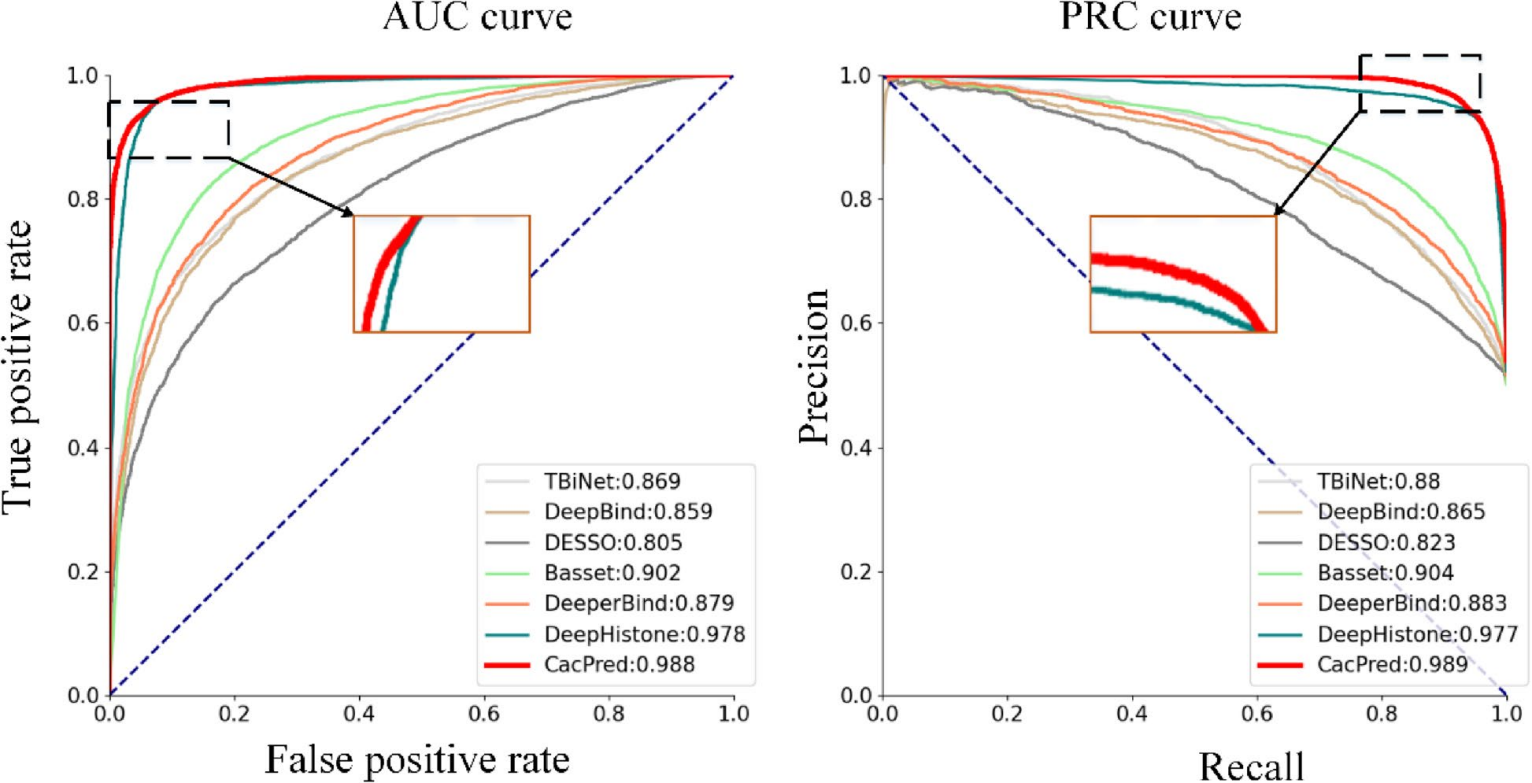
AUC and PRC curves of all models on GSM4072777 dataset

To further test the performance of CacPred, all models are trained and tested on the seven ChIP-nexus datasets in the above way. Based on our results, CacPred outperforms all comparison models (Table 1). CacPred obtains the highest score under precision, recall, F1_score, specificity, ACC, MCC, AUC, and PRC metrics, and achieves the average 0.98, 0.98, 0.97, 0.96, 0.98, 0.97, 0.98, and 0.97 scores, respectively. For the AEMR score, CacPred also obtains the highest score of 2.35, which improves the AEMR by 12.9%. Meanwhile, this paper takes the dataset numbered GSM407277 as an example to show the PRC and AUC curve, the CacPred model achieves the highest PRC and AUC of 0.988 and 0.989 (Fig. 4).

**Table 1:** The average values of the nine metrics

Model	Precision	Recall	F1_score	ACC	Specificity	MCC	AUC	PRC	AEMR
TBiNet	0.74 + 0.02	0.77 ± 0.03	0.75 ± 0.03	0.76 ± 0.03	0.81 ± 0.06	0.55 ± 0.05	0.85 ± 0.02	0.80 ± 0.02	1.46 ± 0.1
DeepBind	0.79 ± 0.01	0.79 ± 0.01	0.79 ± 0.02	0.78 ± 0.01	0.83 ± 0.04	0.58 ± 0.03	0.87 ± 0.02	0.85 ± 0.02	1.51 ± 0.09
DESSO	0.71 ± 0.02	0.71 ± 0.02	0.70 ± 0.01	0.71 ± 0.01	0.72 ± 0.03	0.42 ± 0.03	0.79 ± 0.02	0.81 ± 0.02	1.32 ± 0.05
Basset	0.84 ± 0.01	0.84 ± 0.01	0.84 ± 0.01	0.84 ± 0.01	0.84 ± 0.03	0.68 ± 0.02	0.92 ± 0.01	0.91 ± 0.01	1.73 ± 0.06
DeeperBind	0.79 ± 0.01	0.77 ± 0.02	0.77 ± 0.02	0.77 ± 0.02	0.76 ± 0.1	0.56 ± 0.03	0.88 ± 0.02	0.80 ± 0.02	1.45 ± 0.11
DeepHistone	0.92 ± 0.04	0.91 ± 0.03	0.91 ± 0.03	0.91 ± 0.04	0.94 ± 0.06	0.83 ± 0.07	0.96 ± 0.02	0.96 ± 0.02	2.08 ± 0.1
**CacPred**	**0.98** ± 0.02	0.**98** ± 0.01	**0.97** ± 0.01	**0.96** ± 0.02	**0.98** ± 0.01	**0.97** ± 0.03	**0.98** ± 0.01	**0.97** ± 0.03	**2.35** ± 0.06

### Explaining CacPred model

**Fig. 5:**
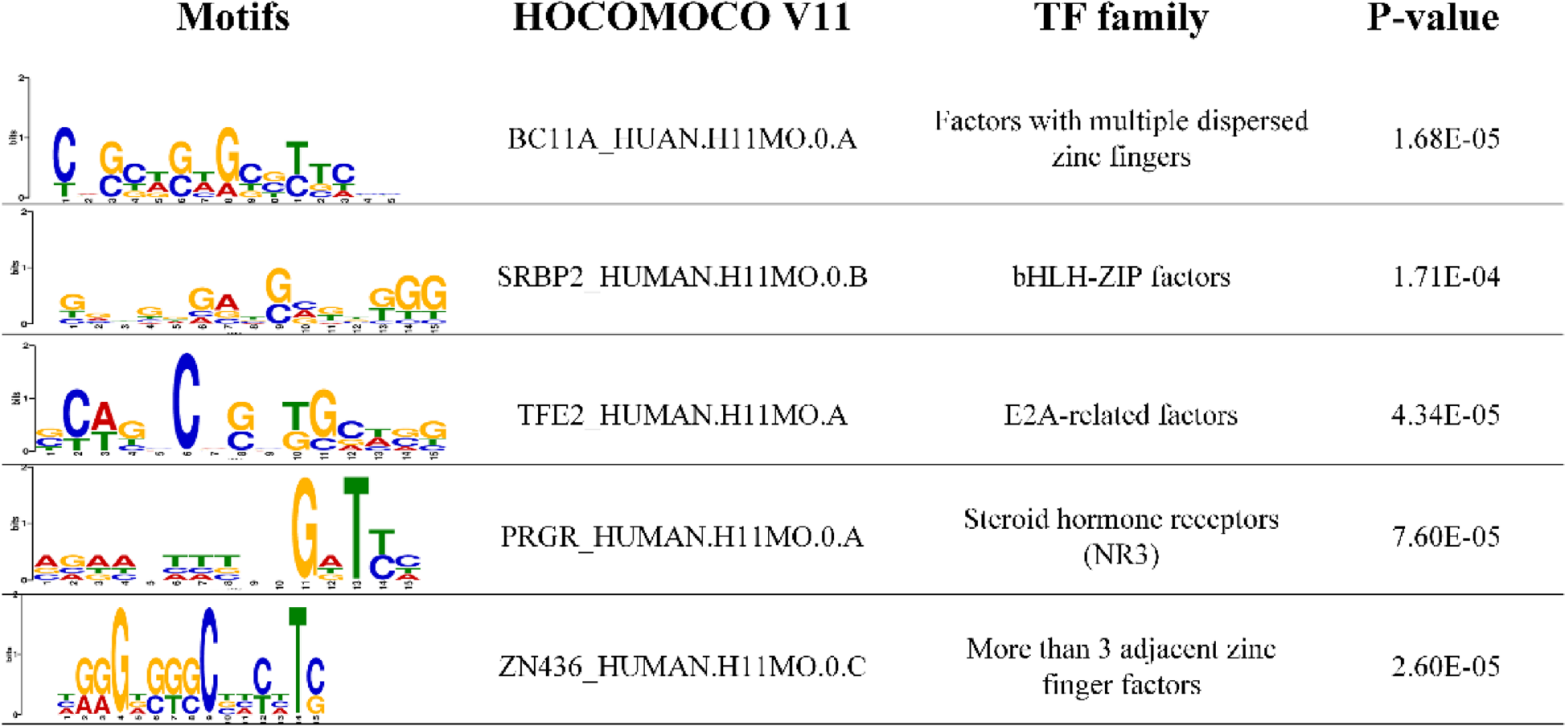
Five significant motifs are found from the wgEncodeEH001833 dataset via the CacPred model

To explain CacPred model, we show features CacPred model learned in a visualized way. CacPred learns features of DNA sequences by the first layer. Each convolutional kernel in the first layer is seen as a motif detector. For each sub-dataset, the forward DNA sequences are fed to the trained CacPred and calculate the outputs of the first layer. Each value of the outputs can represent the importance of each fragment of the input sequence, and the length of each fragment is equal to the width of the convolutional kernel. This study then selects a maximum value of the vector as the activated score (> 0) to obtain the activated sequence (a fragment of the input sequence). After that, this study aligns the set of activated sequences and counts nucleotide occurrences in the set of aligned activated sequences to obtain PWMs. Finally, the underlying TFs’ binding motifs are identified by querying the HOCOMOCO v11 database via the TOMTOM v5.1.0 tool [37]. The matched motifs are significant if their P-values are less than 0.05. The dataset wgEncodeEH001833 is taken as an example (Fig. 5), and five significant motifs are found and visualized by WebLogo [38].

## Discussion

The TF-DNA binding prediction and motif finding are key steps to analyzing and understanding TFs’ functions. This study proposes a cascaded convolutional neural network model (CacPred) for predicting TF-DNA binding and finding motifs. Existing DL models are selected as comparison models, which are compared with CacPred. Our evaluation metrics contain the precision, recall, F1_score, specificity, ACC, MCC, AUC, and PRC, and they are summed as the AEMR score. All comparison models are tested across 790 ENCODE ChIP-seq data and seven ChIP-nexus data. CacPred achieves all the highest metrics, among which the average ACC, MCC, and AEMR are improved by 3.3%, 9.2%, and 6.4% on ChIP-seq data. And the CacPred improves the average ACC, MCC, and AEMR of 5.5%, 16.8%, and 12.9% on ChIP-nexus data. In this study, convolutional algorithms and pooling are applied to all the comparison models, but our proposed method only employs the convolutional algorithms. CacPred achieves the best performance, we reason that the pooling process may lose some sequence information while models are trained. Meanwhile, forward sequence and reverse complementary sequence are fed to CacPred, which provides more sequence information than one of them.

Meanwhile, this study further explores models’ ability to predict TF-DNA binding on cross-cell type ChIP-seq data, and our results show that CacPred also achieved the highest AEMR. To the best of our knowledge, Transcription factor binding preference is easily influenced by different cell types. Considering cross-cell type ChIP-seq data, researchers need to develop new DL frameworks fusing the characterization of different cell types. To interpret CacPred model, some significant motifs are used to show the features CacPred learned, which demonstrates CacPred can automatically learn meaningful features from input sequences.

In this study, CacPred is trained on each sub-dataset, which is suitable for binary classification of each sub-dataset. If we want to apply CacPred to multiple classification on all datasets, CacPred needs to be improved. And owing to the limitation of the width of convolutional kernels, CacPred only can identify TFBS with fixed length. So we should develop convolutional kernels with varied widths to identify TFBS with different lengths. Most of TFs directly bind to the DNA sequences, but TFs sometimes bind indirectly to motifs of other TFs. Assay for Transposase-Accessible Chromatin using sequencing (ATAC-seq) data can simultaneously detect hundreds of TF motif occurrences, but de novo motif discovery tools for ATAC-seq data are lacking [39]. ATAC-seq provides more information to reveal cooperative TF interactions, but the existing models limit the ability to learn motif syntax that promotes TF cooperativity. In recent years, more and more attention has been paid to the expansion of DL methods on graphs. The ideas of CNNs, RNNs, and encoders are applied to graphs, so graph neural networks (GNNs) are developed [40]. GNNs contain graph convolutional networks (GCNs) [41], graph attention networks [42], graph autoencoders [43], etc*.*, which have successes in gene–gene interactions [44]. Considering the successful application of GNNs, this paper infers that GNNs have great potential in motif finding and revealing TFs’ cooperativity.

## Conclusions

This paper introduced CacPred, which utilized cascaded CNN to predict TF-DNA binding from ChIP-seq and ChIP-nexus data. The CacPred significantly improved the AEMR score compared with existing models. And owing to the limitation of the width of convolutional kernels, CacPred only can identify TFBS with fixed length. However, CacPred only employed the convolutional algorithm, and the existing models used convolution and pooling. In light of the experimental results, the existing models may lose some important information in pooling processing. In this study, we demonstrate that CacPred is an effective and feasible model for predicting TF-DNA binding. CacPred is also a potential tool for the other classification tasks in bioinformatics.

## Methods

**Fig. 6:**
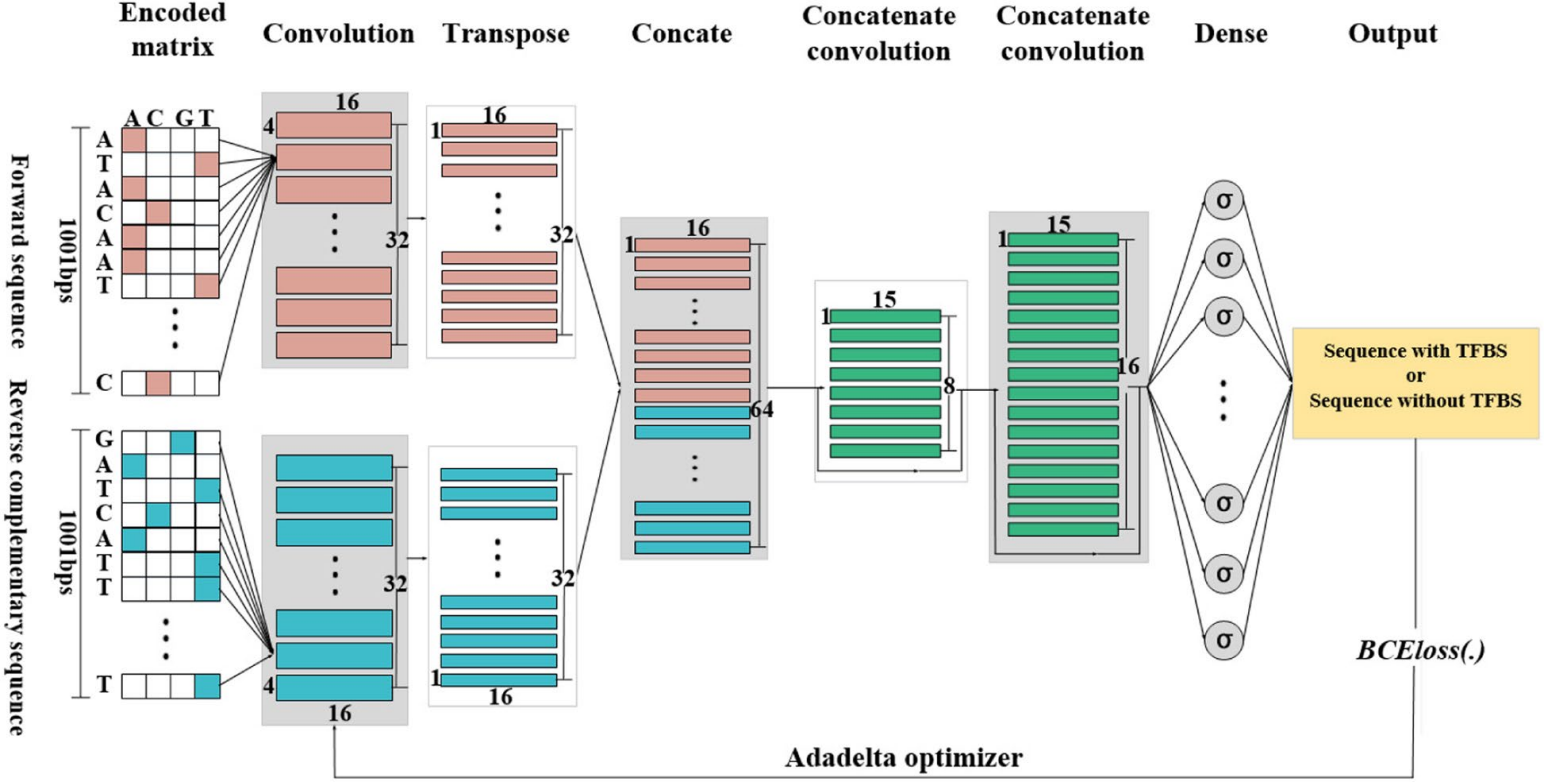
The framework of the CacPred model. The CacPred needs the forward sequences and reverse complementary sequences as inputs, which are encoded into a matrix, respectively. Each red and cadet blue wide-sided rectangle represents a convolution; Each red and cadet blue narrow-sided represents a transposed convolutional algorithm; each green wide-sided rectangle represents a concatenated convolution; σ is the sigmoid function

In this paper, we develop a DL framework named CacPred for TF-DNA binding prediction and finding motifs (Fig. 6). CacPred consists of six layers, i.e., a convolutional layer, a transposed convolutional layer, a combined layer, two concatenated convolutional layers, and a fully connected layer (Fig. 5). CacPred utilized the forward sequences and reverse complementary sequences as inputs, where each input sequence is encoded into a $M = 4 \times 1001$ matrix. The first layer employs two different convolutional layers with 4*16 convolutional kernels to accept the forward sequences and the reverse complementary sequences, and they contain 32 convolutional kernels without sharing parameters respectively. The output of the first layer can be given by formulas (3) and (4).

3 $$C_{11} = {\mathrm{Re}} LU(conv_{11} (M_{1} ))$$

4 $$C_{12} = {\mathrm{Re}} LU(conv_{12} (M_{2} ))$$

where, $M_{1}$ and $M_{2}$ represent forward sequences and reverse complementary sequences encoded matrix, respectively. The $conv_{11} ( \cdot )$ and $conv_{12} ( \cdot )$ represent a convolutional layer of the first layer respectively; ${\mathrm{Re}} LU( \cdot )$ represents the rectified linear unit function; $C_{11}$ and $C_{12}$ are two outputs of the first layer.

The second layer used two transposed convolutional layers, each of them containing 32 convolutional kernels of size 1*16 and a stride of 1. The output of the second layer can be given by formulas (5) and (6).

5 $$C_{21} = trans\_conv_{21} (C_{11} )$$

6 $$C_{22} = trans\_conv_{22} (C_{12} )$$

where $trans\_conv_{21} ( \cdot )$ and $trans\_conv_{22} ( \cdot )$ are transposed convolutional layers; $C_{21}$ and $C_{22}$ represents the outputs of $trans\_conv_{21} ( \cdot )$ and $trans\_conv_{22} ( \cdot )$ respectively.

The third layer is a combined layer, which combines the outputs of the second layer as a matrix, and the output of the third layer can be given by formula (7).

7 $$C_{3} = concatenate([C_{21} ,C_{22} ])$$

The fourth and fifth layers are concatenated convolutional layers with the convolutional algorithm. The fourth layer employs eight convolutional kernels of size 1*15, and the fifth layer employs 16 convolutional kernels of size 1*15. The outputs of the fourth and fifth layers are given by formula (8) and formula (9).

8 $$C_{4} = concatenate([C_{3} ,{\mathrm{Re}} LU(conv(C_{3} ))])$$

9 $$C_{5} = concatenate([C_{4} ,{\mathrm{Re}} LU(conv(C_{4} ))])$$

The sixth layer of CacPred is a fully connected layer with 1,001 neurons and the output can be defined as:

10 $$\hat{y} = sigmoid(\omega \cdot C_{5} + b)$$

$$sigmoid = \frac{1}{{1 + e^{ - x} }}$$

where $\hat{y}$ represents the output of CacPred; $\omega$ represents the weight matrix; $b$ represents the bias.

CacPred selects the Binary Cross Entropy as the loss function (BCELoss):

11 $$BCEloss = - [y \cdot \log (\hat{y}) + (1 - y) \cdot \log (1 - \hat{y})]$$

where $y$ represents the true label; the $\log ( \cdot )$ represents the logarithmic function.

## Supplementary Information

Additional file 1.

## Data Availability

All data analyzed can be downloaded from http://bmbl.sdstate.edu/DESSO/ and http://cistrome.org/db/#/. The accession number of seven ChIP-nexus datasets is listed in the supplementary material Table S2. The source code of the manuscript is available at https://github.com/zhangsq06/CacPred.

## Abbreviations

TFs: Transcription factors
DL: Deep learning
CNN: Convolutional neural network
ChIP-seq: Chromatin immunoprecipitation-sequencing
ChIP-nexus: Chromatin immunoprecipitation experiments with nucleotide resolution through exonuclease, unique barcode, and single ligation
ACC: Accuracy
MCC: Matthews correlation coefficient
AEMR: Area of eight metrics radar
TFBSs: Transcription factor binding sites
PWM: Position weight matrix
SVM: Support vector machine
RNNs: Recurrent neural networks
DBNs: Deep belief networks
AUC: Area under the receiver operating characteristic curve
PRC: Area under the precision-recall curve
STD: Standard deviations
BCELoss: Binary cross entropy loss function
GNNs: Graph neural networks
GCNs: Graph convolutional networks
