# Supplementary material for: CacPred: a cascaded convolutional neural network for TF-DNA binding prediction
Source: BMC Genomics. 2025 Mar 18;26(Suppl 2):264. doi: 10.1186/s12864-025-11399-y (PMC11916463; doi:10.1186/s12864-025-11399-y)
Supplement: Supplementary file 1 — Additional file 1. [file 12864_2025_11399_MOESM1_ESM.docx]

**Supplementary material**

**Table.** S1 The accession number of seven chip-nexus datasets

| Accession no. | GSM4072776 | GSM4072777 | GSM4072778 | GSM4072779 | GSM4087822 | GSM4087823 | GSM4087824 |
| --- | --- | --- | --- | --- | --- | --- | --- |

**Table.** S2 AEMRs of all models on cross-cell type experiment

| EST1 | | | | | | | |
| --- | --- | --- | --- | --- | --- | --- | --- |
| Cell type | TBiNet | DeepBind | DESSO | Basset | DeeperBind | DeepHistone | CacPred |
| GM12878 | 1.96 | 1.97 | 2.01 | 2.09 | 1.61 | 1.97 | 2.21 |
| A549 | 1.97 | 1.93 | 1.89 | 2.03 | 1.61 | 2.01 | 2.28 |
